# Supplementary material for: Just ten percent of the global terrestrial protected area network is structurally connected via intact land
Source: Nat Commun. 2020 Sep 11;11:4563. doi: 10.1038/s41467-020-18457-x (PMC7486388; doi:10.1038/s41467-020-18457-x)
Supplement: Supplementary file 1 — Supplementary Information [file 41467_2020_18457_MOESM1_ESM.pdf]

Supplementary Information for

Just ten percent of the global terrestrial protected area network is structurally connected via intact land

Ward et al.

Correspondence to: m.ward@uq.edu.au

Supplementary Figures

In this supplementary section we provide theoretical and simplified figures to illustrate how the *ConnIntact* metric works. We also provide additional figures for the several sensitivity analyses around different HFP thresholds (i.e., HFP <1 and HFP <10).

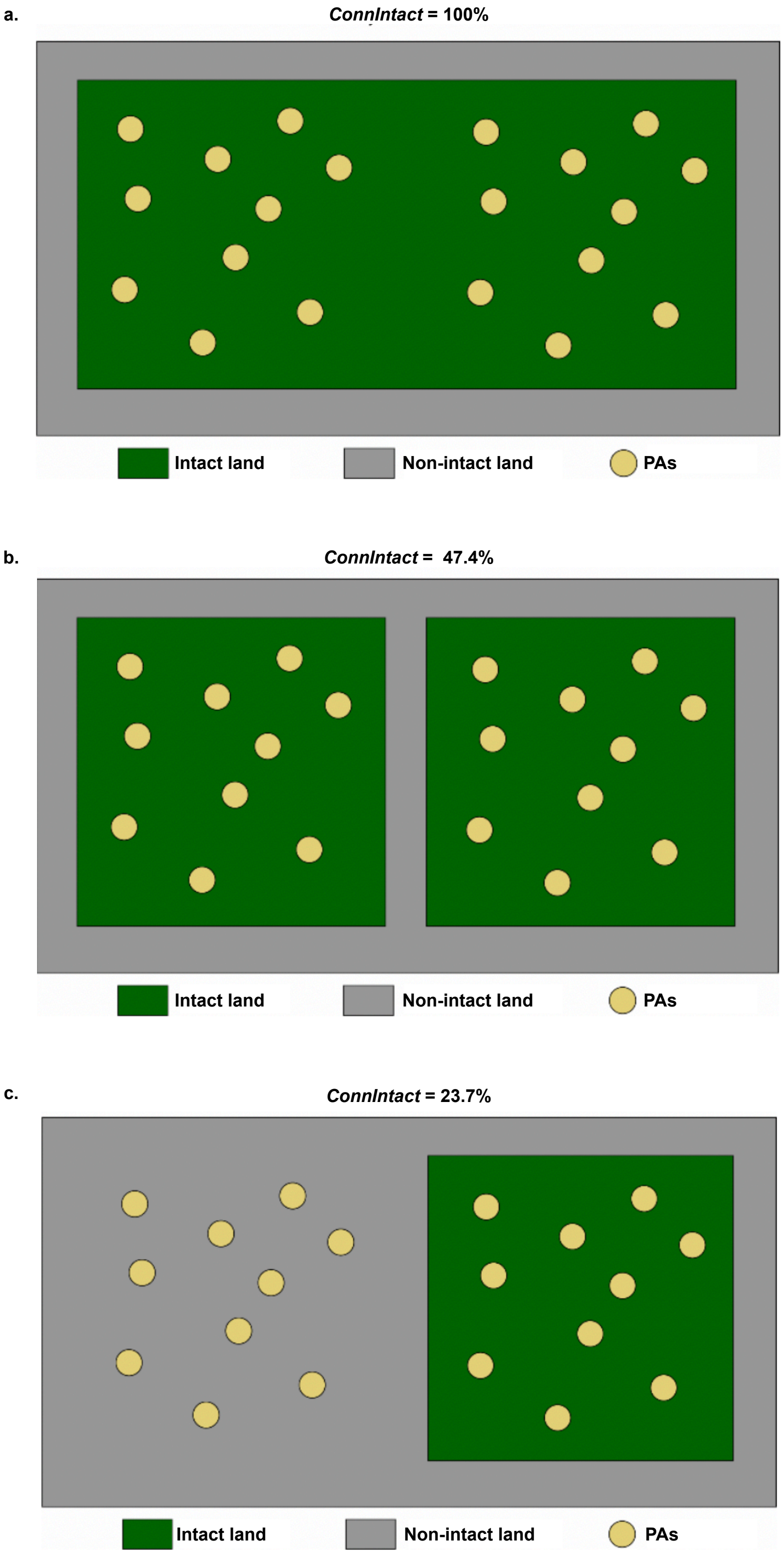
a.
ConnIntact = 100%
Intact land
Non-intact land
PAs
b.
ConnIntact = 47.4%
Intact land
Non-intact land
PAs
c.
ConnIntact = 23.7%
Intact land
Non-intact land
PAs

Supplementary Figure 1. Percentage of the PA network connected by intact lands in three illustrative examples, as quantified through the *ConnIntact* metric used in this study. Supplementary Figure 1a) illustrates an entire country or territory that is covered by one continuous patch of intact land therefore resulting in *ConnIntact* = 100%; Supplementary Figure 1b) illustrates a country or territory that is divided in half by non-intact land, resulting in *ConnIntact* = 47.4%; and Supplementary Figure 1c) is an example of a country or territory maintaining only ten of their PAs within a single patch of intact land, resulting in *ConnIntact* = 23.7%.

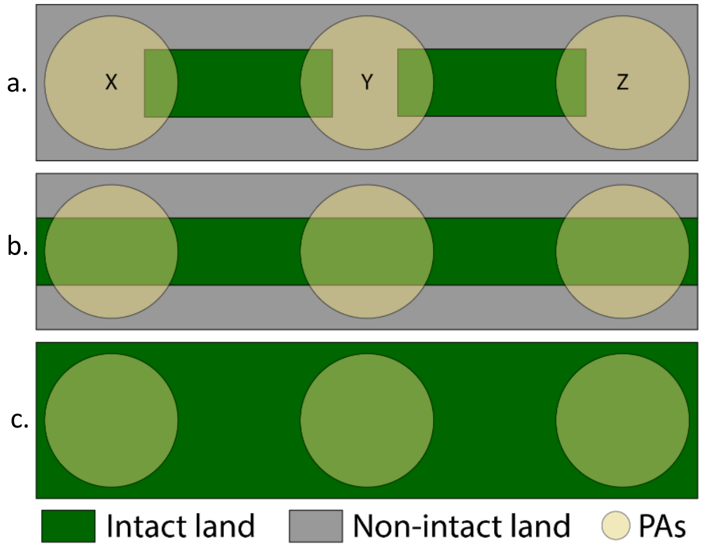

Supplementary Figure 2. Three examples with three PAs with the same *ConnIntact* value (100%) but a different distribution of the intact and non-intact land.

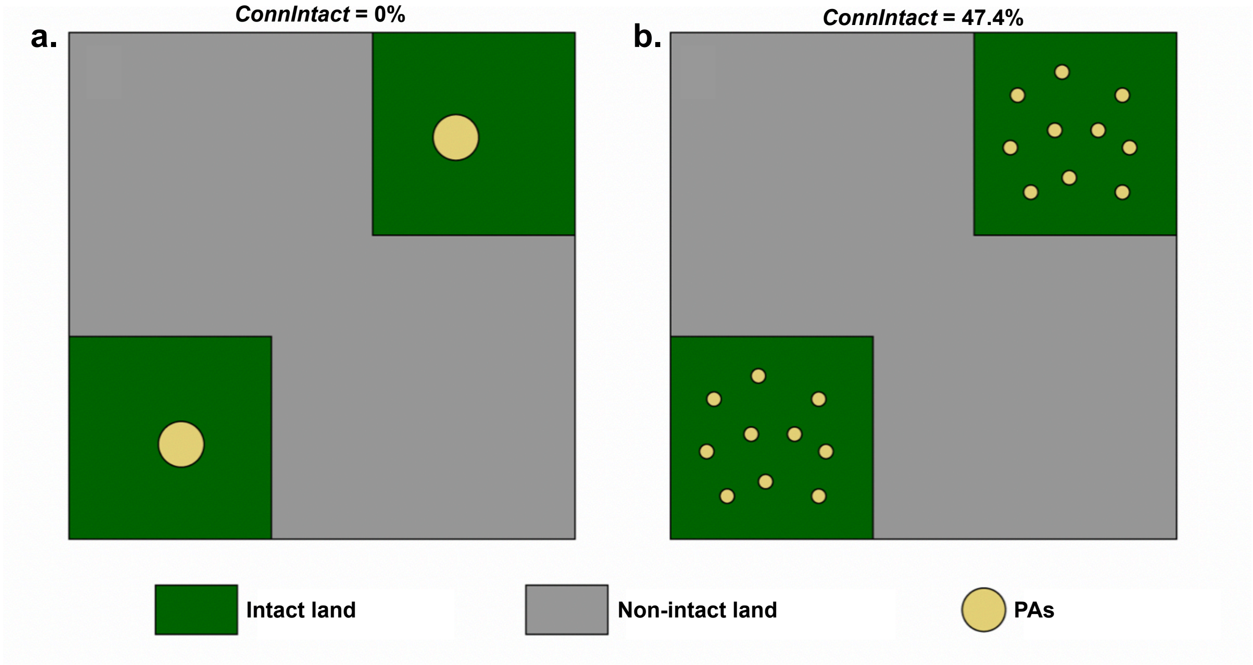

Supplementary Figure 3. Two examples of study areas with the same amount and location of intact land and the same total area under protection. The difference is that in Supplementary Figure 3a there are only two PAs, one in each of the two intact land patches, with an area of 10 km^2 each, while in Supplementary Figure 3b there are 20 PAs with an area of $1km^2$ each, 10 of them found in the lower left intact land patch and the other 10 in the upper right intact land patch in Supplementary Figure 3b.

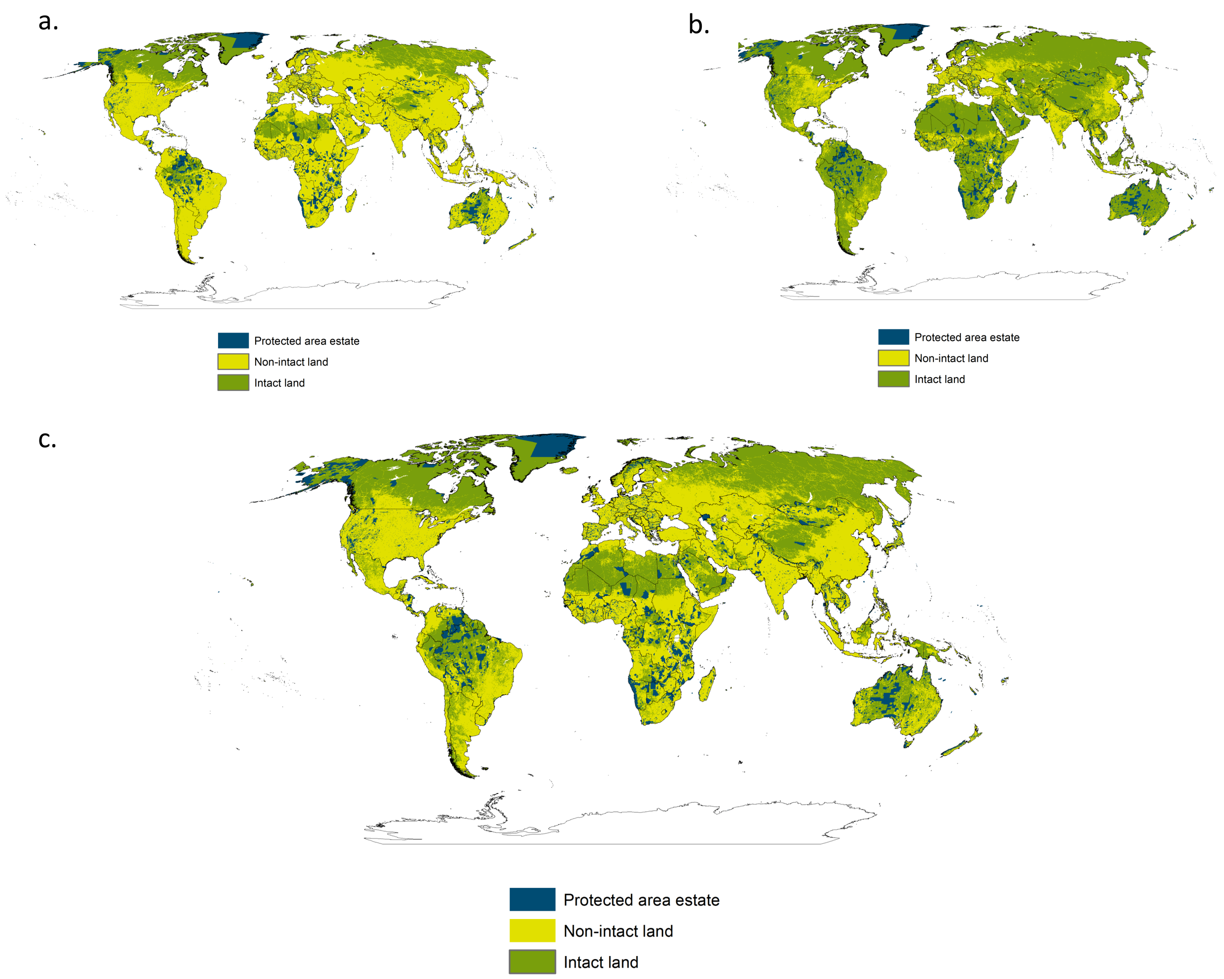

Supplementary Figure 4. Protected area estate. Global non-intact land (yellow), intact land (green), and the spatial distribution of protected area estate (blue) for a) human footprint value <1, b) human footprint value <10, and c) human footprint value <4.

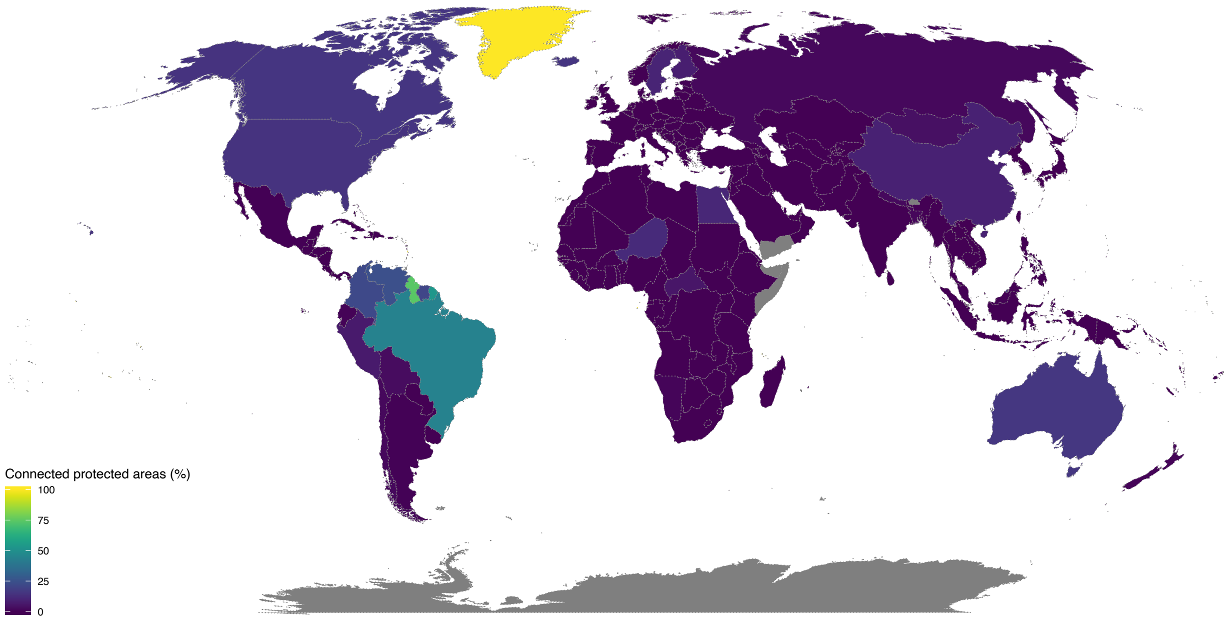

Supplementary Figure 5. Global map showing the proportion of protected areas connected through intact landscapes. Protected areas are regarded as connected if joined by areas with a human footprint value <1.

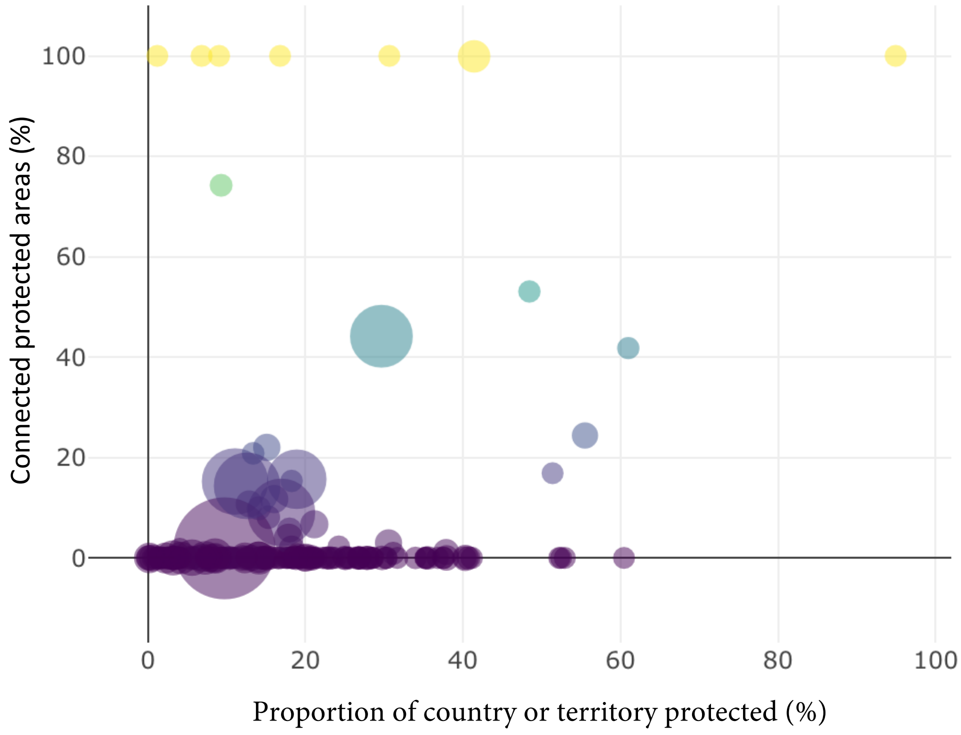

Supplementary Figure 6. Scatterplot showing the relationship between the proportion of land under protection and the proportion of connected protected areas per country or territory. The size of the bubble highlighting the size of the country or territory. Protected areas are regarded as connected if joined by areas with a human footprint value <1.

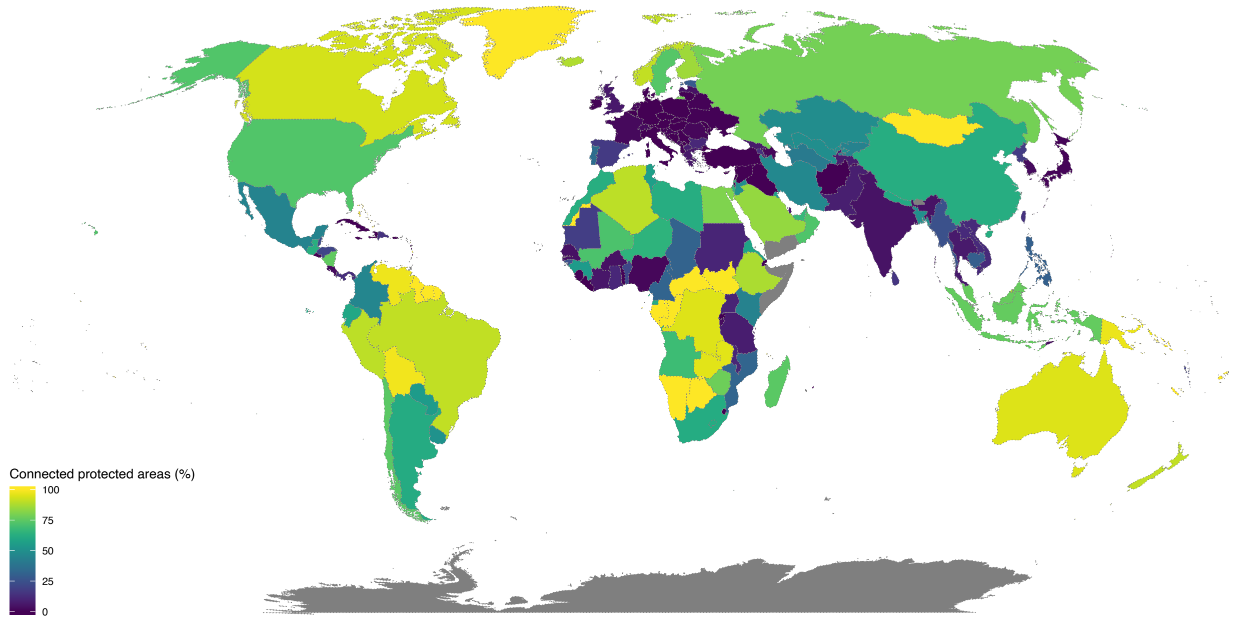

Supplementary Figure 7. Global map showing the proportion of protected areas connected through intact landscapes. Protected areas are regarded as connected if joined by areas with a human footprint value <10.

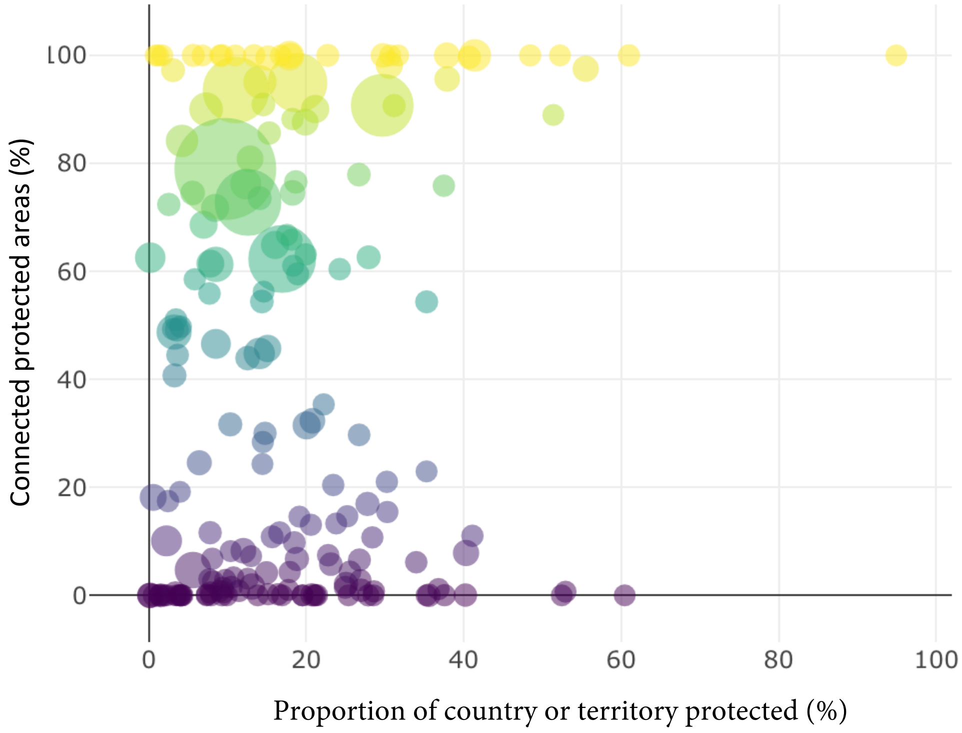

Supplementary Figure 8. Scatterplot showing the relationship between the proportion of land under protection and the proportion of connected protected areas per country or territory. The size of the bubble highlighting the size of the country or territory. Protected areas are regarded as connected if joined by areas with a human footprint value <10.

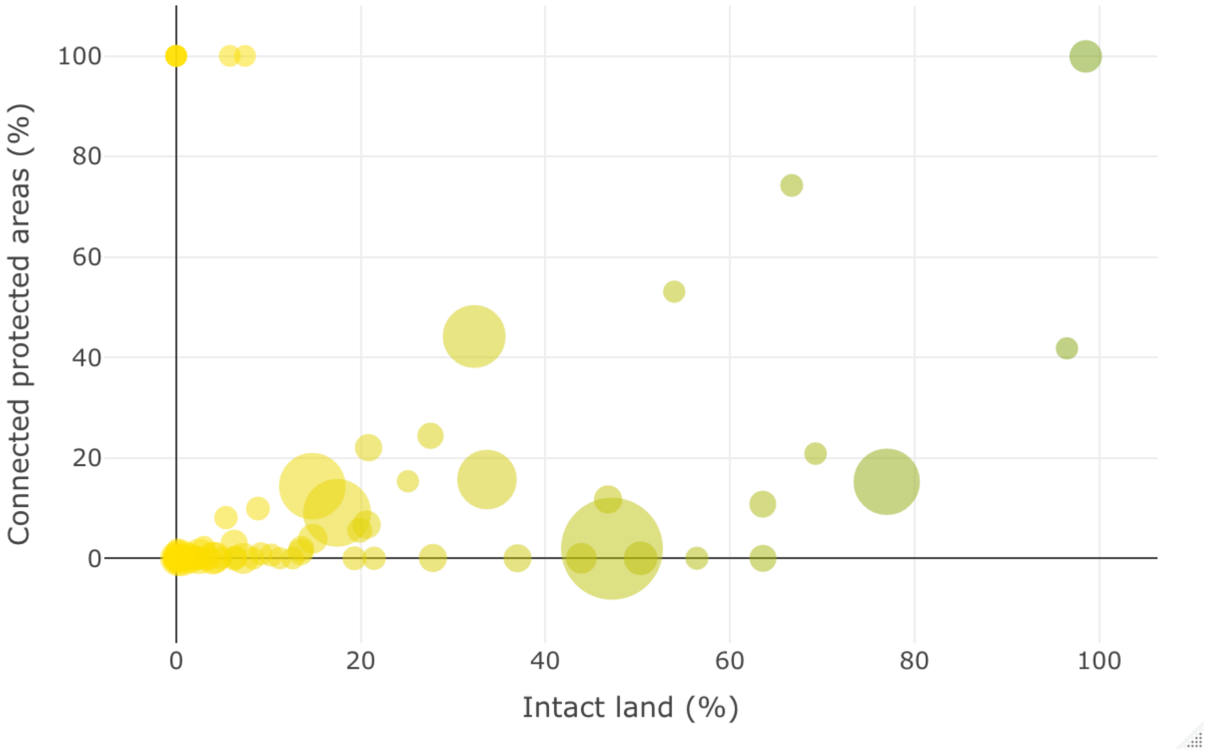

Supplementary Figure 9. Scatterplot showing the relationship between proportion of intact land and proportion of connected protected areas per country or territory. The size of the bubble highlighting the size of the country or territory. Protected areas are regarded as connected if joined by areas with a human footprint value <1.

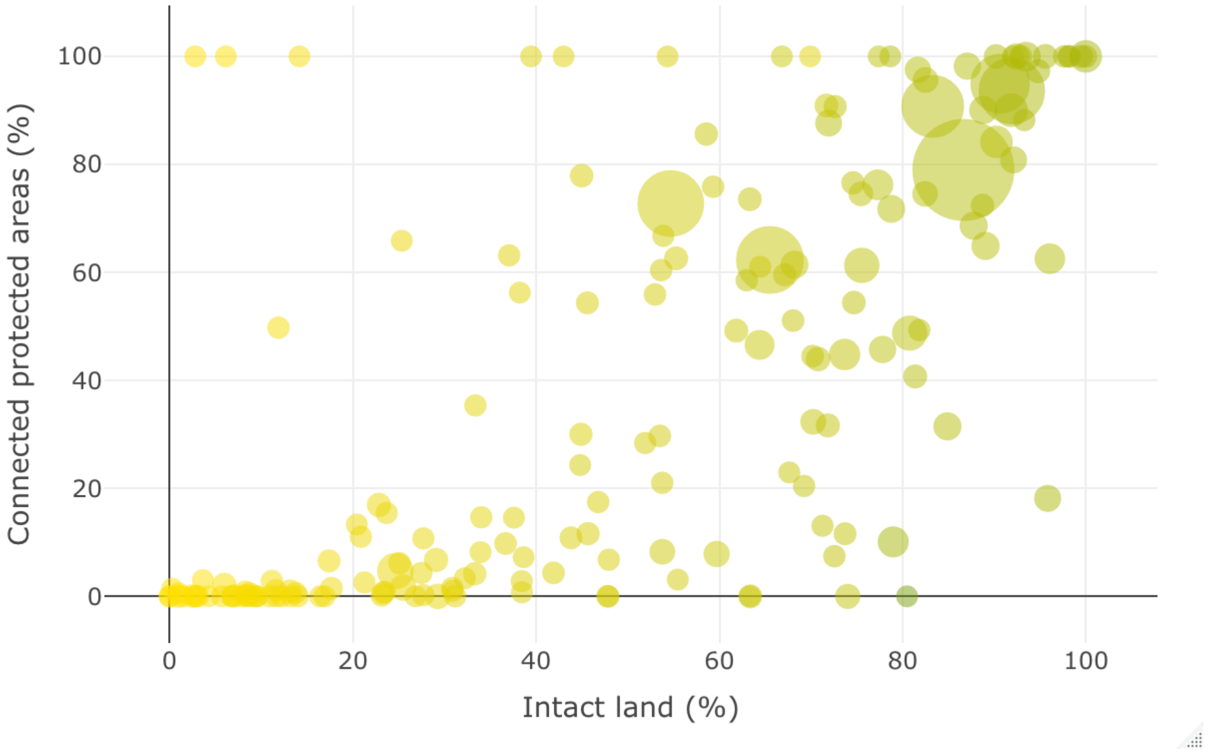

Supplementary Figure 10. Scatterplot showing the relationship between proportion of intact land and proportion of connected protected areas per country or territory. The size of the bubble highlighting the size of the country or territory. Protected areas are regarded as connected if joined by areas with a human footprint value <10.

Supplementary Table 1. Sensitivity analysis of human footprint (HFP) thresholds showing the proportion of protected areas connected through intact pathways (ConnIntact %). All sensitivity analyses were performed considering those PAs with a size of at least 10 km^2.

	Connected protected areas (%)		
Areas of study	ConnIntact (%) HFP <1	ConnIntact (%) HFP <4	ConnIntact (%) HFP <10
Africa	0.1	0.5	34.8
Americas	12.7	14.8	44.4
Asia	2.2	3.2	42.2
Europe	0.1	0.3	2.3
Oceania	15.6	16.8	94.9
Russia*	1.9	4.5	79.0
Global	8.2	9.7	43.5

*Russia was treated as a separate entity because of its large size and large amount of connected land.

Supplementary Table 2. Sensitivity analysis showing the proportion of all land that is intact under different human footprint (HFP) thresholds.

	Percentage of intact land		
	HFP <1	HFP <4	HFP <10
Global	25.0%	41.6%	74.0%
