# Supplementary material for: Just ten percent of the global terrestrial protected area network is structurally connected via intact land
Source: Nat Commun. 2020 Sep 11;11:4563. doi: 10.1038/s41467-020-18457-x (PMC7486388; doi:10.1038/s41467-020-18457-x)
Supplement: Supplementary file 5 — Description of Additional Supplementary Files [file 41467_2020_18457_MOESM5_ESM.pdf]

Description of Additional Supplementary Files

File Name: Supplementary Data 1
Description: Overview of the number of protected areas, area of protected areas, and proportion of connected protected areas under three human footprint thresholds <1, <4, and <10 per country or territory.

File Name: Supplementary Data 2
Description: Overview of the three conditions in area and as a proportion per country or territory.
